# MDR *M. tuberculosis* outbreak clone in Eswatini missed by Xpert has elevated bedaquiline resistance dated to the pre-treatment era

**DOI:** 10.1186/s13073-020-00793-8

**Published:** 2020-11-25

**Authors:** Patrick Beckert, Elisabeth Sanchez-Padilla, Matthias Merker, Viola Dreyer, Thomas A. Kohl, Christian Utpatel, Claudio U. Köser, Ivan Barilar, Nazir Ismail, Shaheed Vally Omar, Marisa Klopper, Robin M. Warren, Harald Hoffmann, Gugu Maphalala, Elisa Ardizzoni, Bouke C. de Jong, Bernhard Kerschberger, Birgit Schramm, Sönke Andres, Katharina Kranzer, Florian P. Maurer, Maryline Bonnet, Stefan Niemann

**Affiliations:** 1 Molecular and Experimental Mycobacteriology, Research Center Borstel, Parkallee 1, 23845 Borstel, Germany; 2 German Center for Infection Research (DZIF), Partner Site Hamburg-Lübeck-Borstel-Riems, Borstel, Germany; 3 Epicentre, Paris, France; 4 Department of Genetics, University of Cambridge, Cambridge, UK; 5 Centre for Tuberculosis, National TB Reference Laboratory, WHO TB Supranational Laboratory Network, National Institute for Communicable Diseases/National Health Laboratory Services, Johannesburg, South Africa; 6 Department of Medical Microbiology, University of Pretoria, Pretoria, South Africa; 7 Department of Internal Medicine, University of Witwatersrand, Johannesburg, South Africa; 8 DST/NRF Centre of Excellence for Biomedical Tuberculosis Research, Faculty of Medicine and Health Sciences, Stellenbosch University, Cape Town, South Africa; 9 South African Medical Research Council Centre for Tuberculosis Research, Division of Molecular Biology and Human Genetics, Faculty of Medicine and Health Sciences, Stellenbosch University, Cape Town, South Africa; 10 SYNLAB Gauting, Gauting, Germany, IML red GmbH, Institute of Microbiology and Laboratory Medicine, WHO Supranational Reference Laboratory of TB, Gauting, Germany; 11 National Tuberculosis Reference Laboratory (NTRL), Ministry of Health, Mbabane, Swaziland; 12 Mycobacteriology Unit, Department of Biomedical Sciences, Institute of Tropical Medicine, Antwerp, Belgium; 13 Médecins Sans Frontières (MSF) - Eswatini, Mbabane, Eswatini; 14 National and WHO Supranational Reference Center for Mycobacteria, Research Center Borstel, Borstel, Germany; 15 London School of Hygiene and Tropical Medicine, London, United Kingdom of Great Britain and Northern Ireland UK; 16 Eppendorf, Institute of Medical Microbiology, Virology and Hygiene, University Medical Center Hamburg, Hamburg, Germany; 17 IRD UMI233/ INSERM U1175/Université de Montpellier, Montpellier, France; 18 Biochemistry & Microbiology, School of Medicine, University of Namibia, Windhoek, Namibia

**Keywords:** Tuberculosis, Multidrug resistance, Resistance evolution, MDR outbreak strains, Diagnostice escape, Treatment escape, Treatment failure

## Abstract

**Background:**

Multidrug-resistant (MDR) *Mycobacterium tuberculosis* complex strains not detected by commercial molecular drug susceptibility testing (mDST) assays due to the RpoB I491F resistance mutation are threatening the control of MDR tuberculosis (MDR-TB) in Eswatini.

**Methods:**

We investigate the evolution and spread of MDR strains in Eswatini with a focus on bedaquiline (BDQ) and clofazimine (CFZ) resistance using whole-genome sequencing in two collections ((1) national drug resistance survey, 2009–2010; (2) MDR strains from the Nhlangano region, 2014–2017).

**Results:**

MDR strains in collection 1 had a high cluster rate (95%, 117/123 MDR strains) with 55% grouped into the two largest clusters (gCL3, *n* = 28; gCL10, *n* = 40). All gCL10 isolates, which likely emerged around 1993 (95% highest posterior density 1987–1998), carried the mutation RpoB I491F that is missed by commercial mDST assays. In addition, 21 (53%) gCL10 isolates shared a Rv0678 M146T mutation that correlated with elevated minimum inhibitory concentrations (MICs) to BDQ and CFZ compared to wild type isolates. gCL10 isolates with the Rv0678 M146T mutation were also detected in collection 2.

**Conclusion:**

The high clustering rate suggests that transmission has been driving the MDR-TB epidemic in Eswatini for three decades. The presence of MDR strains in Eswatini that are not detected by commercial mDST assays and have elevated MICs to BDQ and CFZ potentially jeopardizes the successful implementation of new MDR-TB treatment guidelines. Measures to limit the spread of these outbreak isolates need to be implemented urgently.

## Background

Tuberculosis (TB) is a significant global health problem that is exacerbated by the increasing incidence of drug-resistant TB (DR-TB) [1]. Delayed diagnosis and inappropriate treatment of DR-TB, together with suboptimal implementation of infection control measures, contribute to the emergence and transmission of multidrug-resistant and extensively drug-resistant (MDR, resistant to isoniazid [INH] and rifampicin [RMP]; XDR, MDR strains with additional resistance to a fluoroquinolone [FQ] and one second-line injectable drug [SLID]) *Mycobacterium tuberculosis* complex (Mtbc) strains [2, 3].

Eswatini, a small kingdom bordered by South Africa and Mozambique, has a high burden of TB, MDR-TB and HIV-TB, with an estimated TB incidence of 308 cases per 100,000 and an HIV prevalence of 27% among 15–45 year olds [2, 4]. Between 1997 and 2009, the burden of MDR-TB increased 7-fold from 0.9 to 7.7% among patients newly diagnosed with TB. Among retreatment cases, the proportions of MDR-TB quadrupled from 9.1% in 1997 to 33.9% in 2009 [4, 5]. To address the expanding MDR-TB epidemic, Eswatini has rolled-out the Xpert MTB/RIF, a rapid molecular TB diagnostic with the additional benefit of diagnosing RMP resistance; has expanded access to universal first- and second-line drug susceptibility testing (DST); and has decentralized DR-TB care [6].

A better understanding of the factors contributing and/or driving the MDR-TB epidemic in Eswatini is needed to inform these TB control strategies. This is particularly important because of two unusual findings in this setting. First, we previously showed that one in three MDR-Mtbc strains from the Eswatini 2009–2010 TB drug resistance survey (DRS) harbour the RpoB I491F mutation that is not interrogated by any of the molecular DST (mDST) assays endorsed by the World Health Organization (WHO) [4]. Thus, it is likely that a considerable number of patients with MDR-TB are incorrectly diagnosed as having drug-susceptible TB [4]. This mutation is estimated to account for 0.5% of RMP resistance globally but is frequent in Eswatini because of the clonal transmission of an outbreak strain that has also been described in neighbouring South Africa [7, 8]. By contrast, it is not clear to what extent clonal transmission is responsible for the proportion of RMP resistance that is detected by Xpert (i.e. strains with mutations in the RMP resistance determining region of *rpoB*).

Second, we have demonstrated that some of the RpoB I491F outbreak isolates harbour mutations in *Rv0678*, which encodes the repressor of the MmpS5-MmpL5 efflux pump [7]. If these mutations abolish or reduce the function of Rv0678, an increase in the minimum inhibitory concentrations (MICs) to both bedaquiline (BDQ) and clofazimine (CFZ) via increased efflux of both agents would be expected [9]. Both drugs have been prioritized for the longer MDR-TB regimens in the most recent WHO guidelines and are used in the standardized shorter MDR-TB regimen [10]. Moreover, CFZ is used throughout the entire duration of the recently WHO-endorsed shorter all-oral, BDQ-containing MDR-TB regimen [10, 11]. Clinically significant MIC increases to one or both drugs pre-dating the approval of BDQ would be a serious concern [7].

To investigate the resistance mechanisms, evolution, population structure, and transmission dynamics of MDR/XDR strains in Eswatini in detail, we first performed high-resolution genotyping, including whole-genome sequencing (WGS), for the isolates collected as part of the aforementioned national DRS from 2009 [4, 5]. Moreover, we conducted MIC testing for BDQ and CFZ to explore the potential effects of Rv0678 mutations on the resistance to both agents. Finally, we used WGS to assess the population structure of MDR strains and the frequency of Rv0678 mutations collected in the Nhlangano region between 2014 and 2017.

## Methods

### Strain collections

#### Collection 1

The isolates included in this analysis were collected as part of the TB-DRS in 2009–2010, which has been described in detail elsewhere [4]. Ethical approval was obtained from the Ministry of Health Scientific and Ethics Committee of Eswatini/Swaziland and the Ethics Review Board of Médecins Sans Frontières (MSF). Inclusion in the study was voluntary and after signing of an informed consent form. A total of 412 Mtbc strains were included in this study: 124 MDR, 1 XDR, 267 fully susceptible (DS), and 20 single-drug-resistant (SDR) or poly-drug-resistant (PDR) isolates but non-MDR. For general strain identification, genotyping by 24-loci mycobacterium interspersed repetitive unit-variable number of tandem repeat (MIRU-VNTR) typing and spoligotyping was performed. Additionally, WGS was undertaken for 273 isolates, including all DR isolates and a random selection of DS isolates (122 MDR [two MDR isolates could not be regrown to obtain sufficient DNA for WGS], 1 XDR, 20 SDR/PDR, and 130 DS isolates).

Four genomes from other sources (EMBL-EBI European Nucleotide Archive sequence read archive [study accession IDs: PRJNA393767 [12], PRJEB20942 [13], PRJEB5280 [14], and PRJNA395592 [15]]) were used to demonstrate that the Rv0678 N98D, G121R, and M146T mutations were homoplastic (Additional file 1: Table S1) [16, 17].

#### Collection 2

The genomes of 21 MDR isolates from a study evaluating thin-layer agar (TLA) DST conducted at the Nhlangano Health Centre microbiology laboratory were included to investigate cluster rates and genome characteristics of strains from a more recent period (i.e. between 2014 and 2017, with the majority of isolates from 2015 to 2016; Additional file 1: Table S2). The study protocol was approved by the Institutional Review Board of the Institute of Tropical Medicine (ITM); the Ethics Committee of the University Hospital of Antwerp, Belgium; and the Ministry of Health Scientific and Ethics Committee of Eswatini. Inclusion in the study was voluntary and after signing of an informed consent form. All consecutive patients investigated in Nhlangano (Shiselweni) for presumptive TB, older than 15 years, who had not received TB treatment in the previous month, and consented to be part of the study were included. Further details can be found in the supplementary methods (Additional file 2).

### Laboratory procedures

#### Culture and drug susceptibility testing

Culture and phenotypic DST was performed as stated previously [4, 5]. CFZ and BDQ MICs were measured using the 1% proportion method with the MGIT960 system and the EpiCenter TBeXiST software according to the manufacturer’s instructions (Becton Dickinson, USA). The following concentrations were tested for both drugs: 0.125, 0.25, 0.5, 0.75, and 1 mg/L. Isolates susceptible at 0.125 or resistant at 1 mg/L tests were retested using an extended concentration range (i.e. 0.0312 and 0.0625, or 2 and 4 μg/mL, respectively). BDQ and CFZ MIC of *Rv0678* mutant isolates (*n* = 25; 24 MDR and 1 DS), wild type isolates (*n* = 12; 7 MDR, 2 SDR/PDR, 4 DS; 4 of these were closely related to the *Rv0678* mutant strains [3 gCL 10 strains, 1 S-type strain] and eight were randomly selected) were determined. H37Rv ATCC 27294 was included for quality control in each batch but excluded from the statistical analysis (see Additional file 2 and Additional file 1: Table S3).

#### Classical genotyping

Twenty-four-loci MIRU-VNTR typing and spoligotyping was done using standard approaches as described in supplemental methods previously (Additional file 2). Results were analysed using Bionumerics (version 7.6.3; Applied Maths [bioMérieux, Belgium]). Phylogenetic strain classification and MLVA-MTBC 15-9 nomenclature assignment was performed using the MIRU-VNTR*plus* database [18]. Clusters were defined as two or more Mtbc isolates sharing identical genotyping patterns according to both methods used.

#### Whole-genome sequencing and data analysis

Genomes were sequenced as described previously [19]. The analysis of WGS data was done with bioinformatic pipelines and parameters described before including those for evolutionary studies [19, 20]. A detailed description is provided in the supplement (Additional file 2). WGS data was submitted to the EMBL-EBI European Nucleotide Archive sequence read archive under the study IDs: PRJEB37777 [21], PRJEB6273 [22], PRJEB9680 [23], and PRJEB7281 [24] (Additional file 1: Tables S2 and S4).

### Statistical analysis

Genotyping results were recorded at the molecular typing laboratory and added to the survey database.

Distributions of categorical variables between two comparison groups were compared with Fisher’s exact test. Comparisons of continuous variables were performed with a 2-sample *t* test or Wilcoxon rank-sum test. BDQ and CFZ MICs of mutant and wild type isolates were compared with the Mann-Whitney *U* test. We used an alpha level of 5% for all statistical tests.

## Results

### Population structure, clustering rates, and evolution of *Mycobacterium tuberculosis* complex strains from national drug resistance survey from 2009 to 2010

Based on classical genotyping, the 412 Mtbc strains investigated were classified into nine previously defined phylogenetic lineages and sublineages, which showed no difference in distribution across the four study regions of HhoHho, Lubombo, Manzini, and Shiselweni (Table 1, Additional file 3: Fig. S1 and S2). Cluster (CL) analysis revealed that 278 of the 412 isolates investigated (67%) grouped into 60 MIRU-VNTR/spoligotype clusters (mCL) that comprised between 2 and 34 isolates (Additional file 1: Table S5). The mCL rate was significantly higher among MDR/XDR isolates (90%) compared to DS, SDR, or PDR isolates (58%) (*p* < 0.001). In fact, the two largest clusters mCL6 (*n* = 34, S-type) and mCL15 (*n* = 23, X-type) consisted of MDR isolates only (Additional file 1: Table S5).

**Table 1:** *M. tuberculosis* phylogenetic lineages identified in the isolates analysed

Lineage (sublineage)	Non-MDR/DS (***n*** = 287)		MDR^**1**^ (***n*** = 125)		Overall (***n*** = 412)	
	No.	%	No.	%	No.	%
**Beijing**	82	28.6	6	4.8	88	21.4
**Delhi/CAS**	3	1.1	0	0.0	3	0.7
**East African/Indian**	18	6.3	15	12.0	33	8.0
**Euro-American (Haarlem)**	13	4.5	6	4.8	19	4.6
**Euro-American (LAM [Latin-American/Mediterranean])**	72	25.1	5	4.0	77	18.7
**Euro-American (S-type)**	10	3.5	46	36.8	56	13.6
**Euro-American (Swaziland 1)**	11	3.8	0	0.0	11	2.7
**Euro-American (Swaziland H37Rv like)**	18	6.3	9^1^	7.2	27	6.6
**Euro-American (URAL)**	1	0.4	0	0.0	1	0.2
**Euro-American (X-type)**	43	15.0	38	30.4	81	19.7
**Euro-American superlineage**	16	5.6	0	0.0	16	3.9

^1^Includes one XDR strain

To further investigate the phylogeny and degree of clonality of MDR-Mtbc strains and to identify potential success markers on the genome level (e.g. potential compensatory mutations [19]), we performed WGS of a subset of 273 isolates (Additional file 3: Fig. S3). These included all drug-resistant isolates (122 MDR, 1 XDR, and 20 SDR/PDR) and a random selection of 130 DS isolates.

**Fig. 1:**
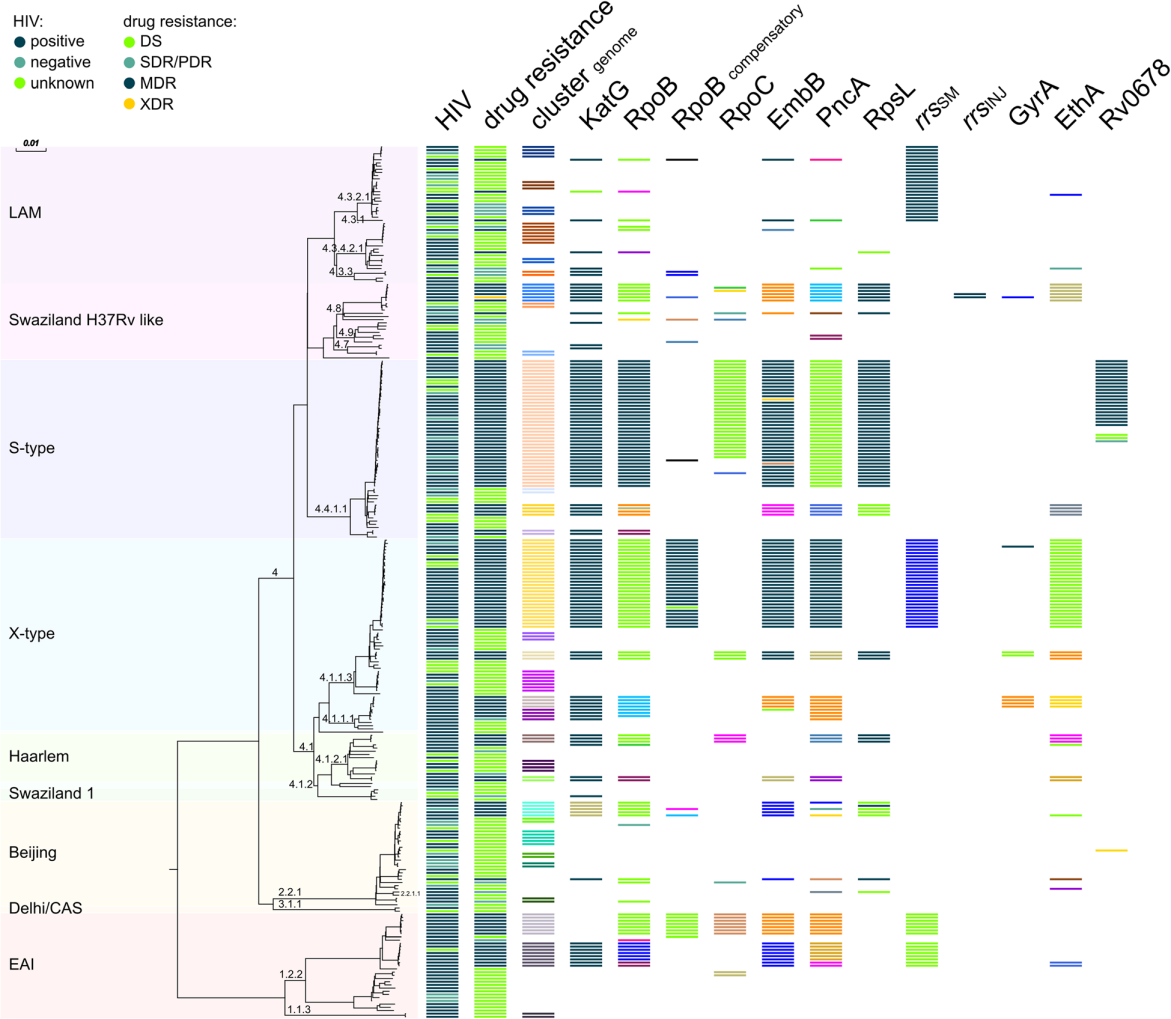
Phylogenetic diversity of 273 *M. tuberculosis* complex isolates from Eswatini. Data are presented in a maximum likelihood tree (MLT) based on 12,062 SNP positions. Phylogenetic lineages are displayed. Cluster genome: isolates in one cluster share the same colour. Occurrence of resistance mediating and putative compensatory mutations in RpoB and RpoC are highlighted; identical mutations share the same colour

The genome-based phylogeny confirmed the strain classification based on classical genotyping (Fig. 1). WGS-based cluster analysis (gCL) based on a 12 SNP distance revealed that 173 of the 273 isolates were in 32 clusters ranging in size from 2 to 40 isolates (Fig. 1 and Additional file 1: Table S5). With 95%, the cluster rate among MDR/XDR was very high (117/123 analysed MDR/XDR isolates), whereas the rate of SDR/PDR/DS was only 37% (56/150 analysed SDR/PDR/DS isolates). Isolates of the two largest clusters (i.e. gCL3 [*n* = 28, X-type] and gCL10 [*n* = 40, S-type]) represented 55% of the M/XDR isolates investigated (Additional file 1: Table S5).

Bayesian coalescent analysis suggested that the most recent common ancestors of each of the 11 MDR genome clusters, respectively, emerged approximately 6.9 to 20.5 years prior to the Eswatini DRS in 2009–2010 (Table 2). The two largest MDR clusters, gCL10 and gCL3, likely emerged 17.2 years (12.4–22.6 years 95% HPD) and 20.5 years (13.6–24.5 95% HPD) prior to the DRS.

**Table 2:** Emergence of main MDR genome clusters and association with putative compensatory mutations

gCL (***N*** isolates)	Lineage	Cluster age [years] (95% HPD)	Isolates with putative compensatory mutation
1 (8)	EAI	15.8 (9.7–22.3)	0 (0%)
3 (28)	X-type	20.5 (13.6–24.5)	28 (100%)
6 (7)	EAI	16.5 (11.2–22.7)	7 (100%)
8 (5)	Beijing	7.8 (4.7–11.3)	2 (40.0%)
9 (4)	S-type	6.9 (3.5–11.2)	0 (0%)
10 (40)	S-type	17.2 (12.4–22.6)	33 (82.5%)
12 (3)	Haarlem	7.3 (3.0–12.4)	3 (100%)
16 (6)	Swaziland H37Rv like	10.8 (6.6–15.4)	3 (50.0%)
19 (3)	X-type	12.8 (5.0–18.3)	3 (100%)
21 (3)^1^	X-type	18.4 (11.4–22.3)	0 (0%)
30 (4)	X-type	7.2 (3.0–8.4)	0 (0%)

*HPD* highest posterior density

^1^MDR isolates only

### Resistance mediating and compensatory mutations

One hundred twenty-three isolates had variations in KatG, 129 in RpoB, 113 in EmbB, 120 in PncA, 67 in *rrs* (streptomycin [SM]), 8 in GyrA, and 65 had variations in RpsL (Fig. 1 and Additional file 1: Table S6). The most common mutations found in *katG* and *rpoB* were the low fitness cost mutations resulting in S315T amino acid substitution (84%, 117/139 INH-resistant isolates) and S450L (47%, 60/127 RMP-resistant isolates; S531L *E. coli* numbering), respectively. The second most frequent mutation in RMP-resistant isolates was RpoB I491F (31%, 40/127 RMP-resistant isolates; I572F *E. coli* numbering) (Additional file 1: Table S6). Twenty-five isolates were found to have mutations in Rv0678, a gene involved in resistance to BDQ and CFZ (one Beijing isolate, and 24 gCL10 MDR outbreak cluster isolates, see below and Additional file 1: Table S6).

The analysis of compensatory mutations revealed that 45 isolates had a variant in RpoB, 11 in RpoA, and 52 in RpoC (Additional file 1: Table S6). Among gCL and non-gCL RMP-resistant isolates, 73% (86/118) and 44% (4/9) harboured putative compensatory mutations, respectively.

We then correlated the detected resistance mutations with the gCL classification. This underlined the high clonality of the outbreak cluster isolates defined by the cluster analysis. For example, all 40 gCL10 isolates carried the same mutations in KatG (S315T), RpoB (I491F), EmbB (M306I), and PncA (H51D), including a subgroup of 24 isolates with additional mutations in Rv0678 (Additional file 1: Table S3). All 28 gCL3 isolates shared the same mutations in KatG (S315T), RpoB (S450L), EmbB (M306I), and PncA (R154G) (Additional file 1: Table S7).

**Fig. 2:**
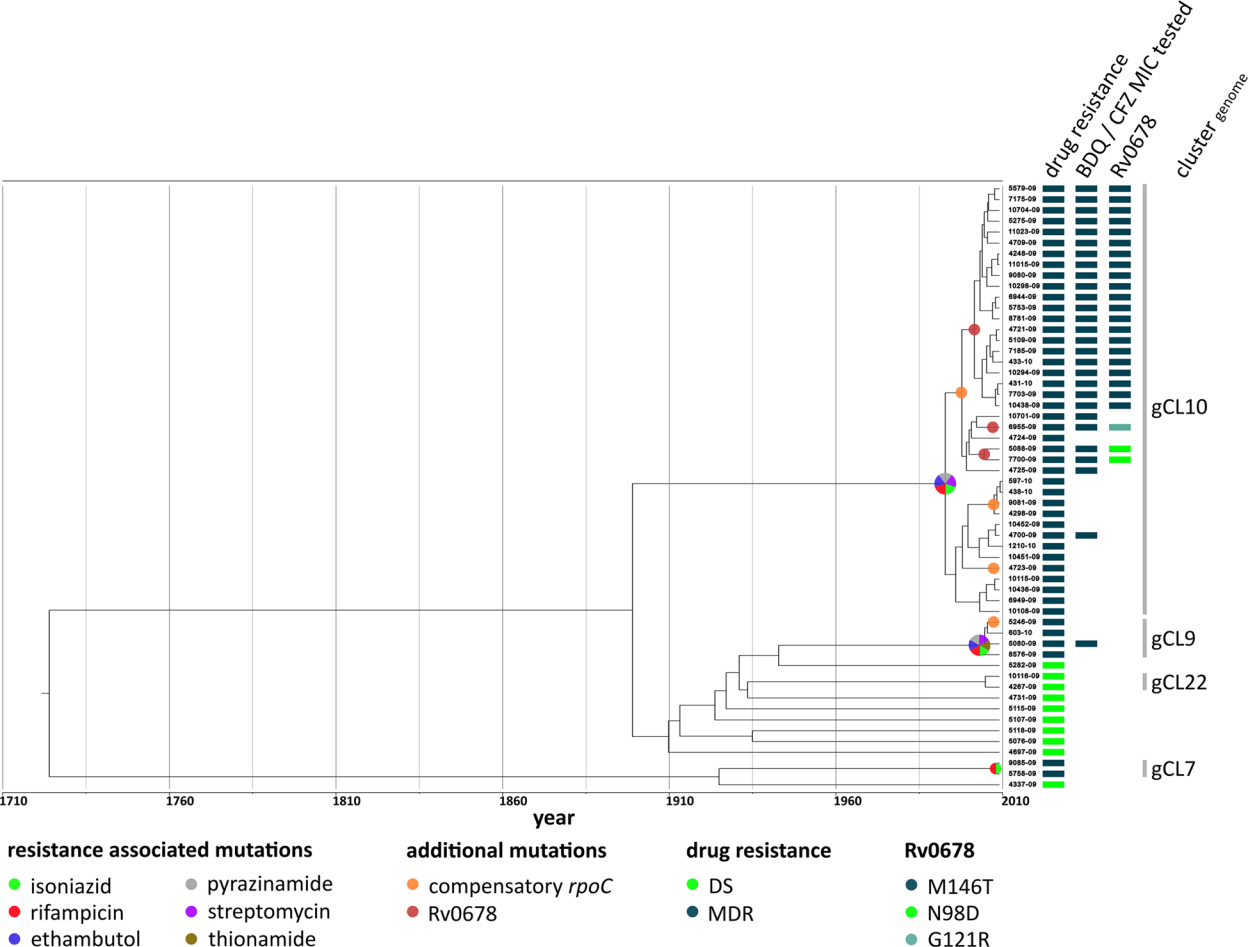
Maximum clade credibility tree of the S-type lineage. gCL10, the largest MDR cluster in Eswatini, has been circulating for 17.2 years prior to the DRS conducted in 2009–2010. Three different Rv0678 mutations occurred within this cluster. The M146T mutation was the most common (*n* = 21) and likely arose 6.7 years prior to the DRS. The N98D (*n* = 2) and G121R (*n* = 1) must have evolved within 5.5 and 7.8 years prior to the DRS, respectively. Isolates, for which BDQ and CFZ MICs were measured, are highlighted (Fig. 3 and Additional file 1: Table S3)

Based on Bayesian coalescent analysis, we calculated a dated maximum clade credibility tree (MCCT) for the S-type lineage and mapped the identified drug resistance-associated mutations to the MCCT (Fig. 2). This analysis showed that isolates of gCL10 acquired resistance to all four first-line drugs and streptomycin (SM) before they started spreading in the community. Isolates of gCL10 acquired further variants in Rv0678 and compensatory mutations in RpoB and RpoC (Additional file 1: Table S6).

### Phylogenetic analysis of Rv0678 mutations and their impact on clofazimine and bedaquiline minimum inhibitory concentrations

We observed four different Rv0678 mutations in collection 1 (Additional file 1: Table S6). A single DS Beijing isolate harboured an A110V mutation, whereas the remaining three occurred in 24 of the 40 gCL10 MDR outbreak cluster isolates with the RpoB I491F mutation (21 isolates with Rv0678 M146T, 2 isolates with Rv0678 N98D, and 1 isolate with Rv0678 G121R; Fig. 2). The three mutations associated, i.e. Rv0678 G121R, N98D, and M146R, with lineage 4 (S-type) strains in the gCL10 MDR cluster evolved independently 5-8 years prior to the DRS (Additional file 1: Table S7). Further, all three mutations were found in other strain collections and importantly other Mtbc lineages such as lineage 4 (Haarlem sublineage) and lineage 2 (Beijing) (Additional file 1: Table S1). This homoplasy (i.e. independent acquisition of one trait/mutation in distinct phylogenetic backgrounds) is often a sign of positive selection [26].

**Fig. 3:**
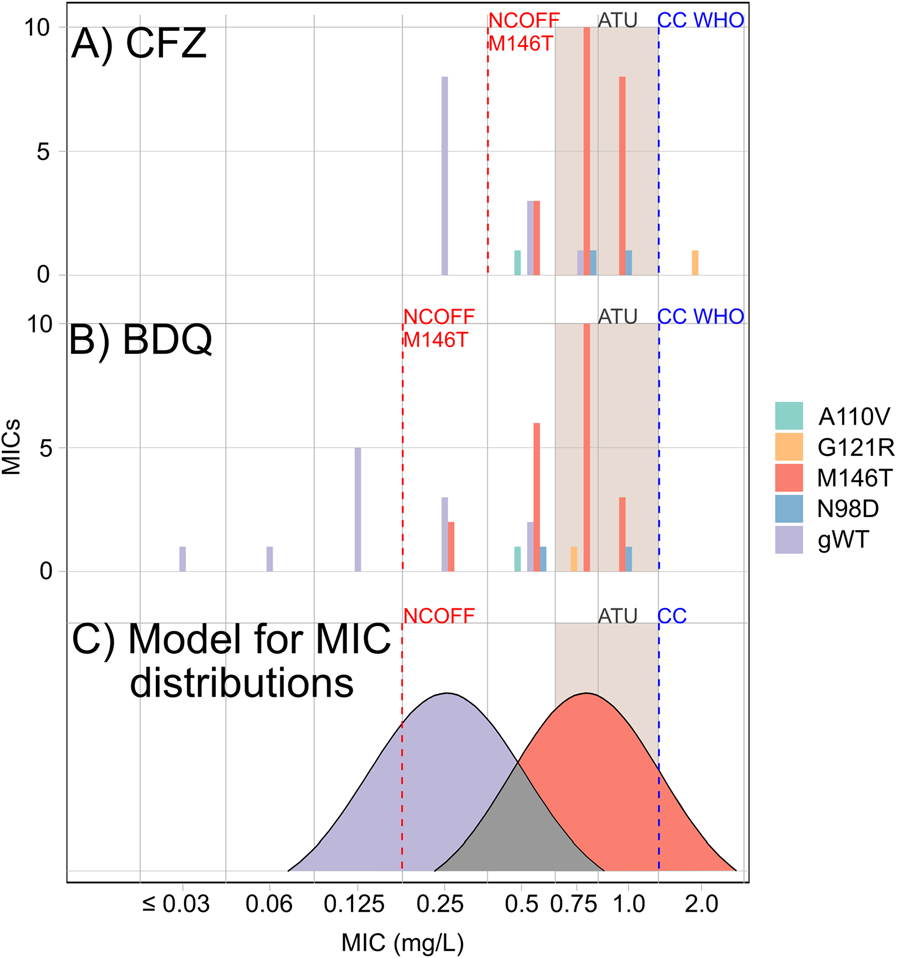
Effect of Rv0678 mutations on BDQ and CFZ MICs. The MICs for the DS Beijing mutant with the Rv0678 A110V mutation were compared with those of 24 gCL10-outbreak MDR isolates with either the Rv0678 N98D, G121R, or M146T mutations (Fig. 2). The NCOFFs for the Rv0678 M146T distributions for CFZ (part **a**) and BDQ (part **b**) were set using the eyeball method, and a corresponding idealized representation of the MIC distributions of the gWT and Rv0678 M146T populations for both drugs (and their area of overlap in grey) was prepared for illustrative purposes (part **c**) [25]. The MIC increases for M146T relative to gWT isolates were statistically significant for both CFZ and BDQ. The Rv0678 G121R and A110V mutations could not be evaluated statistically as the mutations only appeared once

To investigate their impact on resistance levels, we measured the BDQ and CFZ MICs for all Rv0678 mutants in comparison with genotypically wild type (gWT) control isolates that did not harbour any mutations in Rv0678, three of which were gCL10 strains without Rv0678 mutations (see the “Methods” section, Fig. 3A, B). The 21 gCL10 outbreak isolates with the Rv0678 M146T mutation had significantly elevated MICs for BDQ and CFZ relative to gWT strains (*p* < 0.001 for both drugs; Fig. 3A, B) as well as compared to gWT gCL10 strains (*p* < 0.05). This was also apparent when comparing the modes of the MIC distributions of the M146T mutation, which were three times higher than those of gWT isolates (i.e. 0.75 mg/L vs. 0.25 mg/L), although both distributions overlapped considerably. In fact, even if areas of technical uncertainty (ATUs, as outlined in Additional file 2) were introduced at 0.75–1 mg/L for both drugs, a proportion of these mutants would still be classified as susceptible because of the technical variation in MIC testing (i.e. the non-wild type cut-off value (NCOFF) for CFZ was 0.25 and 0.125 mg/L for BDQ, which would have to be tested to reliably detect this mutation, Fig. 3A–C).

The MIC increases for both drugs were also statistically significant for the two strains with the Rv0678 N98D mutation (BDQ: 100% of MICs ≥ 0.5 mg/L, *p* value 0.03297; CFZ: 100% of MICs ≥ 0.5 mg/L, *p* value 0.01099). The BDQ and CFZ MICs for the strain with Rv0678 mutation G121R were 2 mg/L and, thus, fell into the resistant range but could not be evaluated statistically as this mutation was observed only once. Similarly, statistical analyses could not be performed for the Rv0678 A110V mutation, which tested susceptible to CFZ and BDQ with MICs of 0.5 mg/L.

### Population structure of multidrug-resistant *Mycobacterium tuberculosis* complex strains from the Nhlangano region between 2014 and 2017

The genome-based cluster analysis of MDR-Mtbc strains from the TLA study revealed a high cluster rate (20 out of 21) with isolates from four genome clusters already found in collection 1 (Additional file 1: Table S2). Isolates of the two dominant outbreaks now accounted for seven (gCL3, 33%) and nine (gCl10, 43%) of the 21 MDR strains investigated (because of the different sampling strategies, these frequencies could not be directly compared with those from the DRS). Six of the nine gCl10 isolates (67%) shared the aforementioned Rv0678 M146T mutation.

## Discussion

This study confirmed that clonal transmission of particular MDR-Mtbc outbreak clones has been the main driver of the MDR-TB epidemic in Eswatini. In fact, the two dominant gCL3 and gCL10 outbreak strains had already acquired resistance to all first-line drugs and SM when they began to circulate approximately 30 years ago. Our data show that in the DRS from 2009, the most frequent outbreak strain (gCL10) accounted for 30% of MDR isolates. Thereafter, it continued to spread, as shown by our analysis of collection 2, to reach 56% in the latest national DRS from 2017 to 2018 as outlined in the WHO Global tuberculosis report 2019 [1, 27]. Unfortunately, we did not have access to the genomes of the strains from this latest DRS, which would have provided an even clearer picture of the MDR-TB epidemiology in Eswatini. Nevertheless, our results supported a number of notable conclusions.

Although all WHO-endorsed mDST assays do not interrogate the RpoB I491F that is common to all gCL10 strains, we found that these strains all shared the KatG S315T mutation that is covered by all WHO-endorsed line probe assays for first-line drugs. Therefore, isolates with this KatG mutation could be prioritized for reflex testing with assays capable of detecting RpoB I491F to inform treatment decisions [28]. To this end, we have started to implement the targeted next-generation sequencing (NGS) assay Deeplex-MycTB in Eswatini, which not only covers all major resistance genes to traditional first- and second-line drugs as well as Rv0678 but also enables genotyping based on the spoligotype and phylogenetically informative mutations from primary specimens, e.g. sputum [29, 30]. Such a triage approach will, however, inevitably be slower than diagnosing RMP resistance testing with Xpert, which may provide a continued evolutionary advantage to gCL10 [6]. Accordingly, a prospective investigation of the current epidemiological scenario related to the spread of the gCL10 outbreak strains is planned.

In addition to being resistant to all first-line drugs, the gCL10 clone acquired multiple Rv0678 mutations that correlate with elevated MICs to BDQ and/or CFZ and have been circulating since 2009 (and likely several years earlier, as indicated in the dated phylogenies). The evolutionary pressures for this phenomenon are not clear. BDQ was not used in Eswatini at the time of the first DRS and was not introduced on a larger scale until 2015 in the first clinical trials [31]. Given that CFZ featured in an MDR-TB treatment guideline from 2008 [32], we cannot exclude that individual patients were treated with CFZ in Eswatini or neighbouring countries. Alternatively, antifungal azoles, which are commonly used in sub-Saharan Africa, may be responsible [33].

The dominant mutation Rv0678 M146T shared by 21 (53%) gCL10 isolates correlated with MICs to both drugs that were approximately three times higher than for gWT isolates (62% of MICs ≥ 0.75 mg/L). To prove conclusively whether M146T is causative of this increase would require allelic exchange experiments, which were beyond the scope of this study. It is not clear whether these particular MIC increases are clinically significant given that no pharmacokinetic/pharmacodynamic targets for the efficacy of BDQ or CFZ have been set by the European Committee for Antimicrobial Susceptibility Testing (EUCAST) or WHO [9, 34]. Instead, conservative clinical breakpoints (CBs), as defined by EUCAST, have been set that correspond to the likely epidemiologic cut-off values (ECOFFs) of 1 mg/L [9, 35]. Consequently, it was assumed that any MIC increase above the ECOFF would result in worse treatment outcomes, until strong evidence to the contrary is presented. In such a case, the CB could be raised above the ECOFF and/or a second, higher CB could be introduced to define a range of susceptibility at increased exposure (previously known as “intermediate”) [35, 36]. This does not mean that isolates with MICs at or below the current CB are necessarily susceptible given that they could harbour a resistance mechanism that results in only modest MIC increases. To minimize the misclassification of such mutations, an ATU for both drugs could be set and the concentration below the CB would have to be tested, as opposed to just the CB, as currently recommended by WHO (Fig. 3) [36]. Indeed, Nimmo et al. have made a similar proposal for 7H11 DST [33].

In practice, it will be impossible to study the effect of all Rv0678 variants on resistance levels comprehensively, considering the large number of possible mutations with likely heterogeneous effects on MICs (i.e. depending on whether they abolish the function of the repressor completely or not, which is further complicated by the fact that mutations can only confer elevated MICs in strain backgrounds with a functional efflux pump) [37–41]. Moreover, simply correlating clinical outcomes with MICs is complicated by the overlap between MIC distributions. Compared with resistance mechanisms that confer marked MIC increases (e.g. delamanid), BDQ and CFZ will, therefore, pose significant challenges to regulatory agencies and diagnostic laboratories [9, 39, 42]. Nevertheless, the evidence is mounting that particular Rv0678 mutations emerge in patients on failing regimens, confer elevated MICs to both drugs, and adversely affect clinical outcomes [37–40, 43–46]. Indeed, Eswatini will likely provide a unique opportunity to study the effect of a single Rv0678 variant on clinical outcomes because the M146T mutation is so frequent in this setting, which would provide invaluable data regarding the efficacy of the all-oral shorter MDR-TB regimen.

A limitation of this study is selection bias, due to exclusion of some (mostly poorly growing) isolates from the genotyping analysis for the strains from the TB-DRS in 2009–2010. Because the excluded isolates were mainly DS, our findings about the MDR-TB clusters are unlikely to be biased significantly. But, any comparison between DS and MDR-TB results has to be interpreted with caution. Another limitation is the fact that the majority of the isolates were from 2009, and the overall number of MDR-Mtbc strains likely represents only about 30% of all MDR-Mtbc strains emerging in that period in Eswatini [4, 5, 47]. This is even more relevant for our second collection that sampled MDR-Mtbc strains from just one region that represents a small fraction (below 5%) of the MDR-Mtbc strains in that period [47]. Still, our analysis of collection 2 isolates and the recently presented data from the recent DRS survey performed in years 2017–2018 underline the current importance of RpoB I491F MDR outbreak cluster isolates in Eswatini [1, 27], but prospective more comprehensive data are needed to detail the ongoing MDR-TB transmission in the country.

## Conclusions

From a public health perspective, our findings that transmission and increasing prevalence of highly resistant strains plays a key role in Eswatini’s expanding MDR-TB epidemic underline the urgent need to prioritize breaking transmission chains in the country. This, in turn, means focusing on interventions, such as improving diagnosis of MDR-TB, including RpoB I491F mutants, tracing of MDR-TB patient contacts, implementing effective infection control measures within healthcare facilities and communities, and careful epidemiological monitoring of the spread of MDR outbreak clones on a regional scale and beyond. Moreover, the effect of the Rv0678 M146T mutation on treatment outcomes with BDQ- and CFZ-containing regimens and enhanced transmission rates should be studied as a priority, including using the most recent DRS data.

Our study raises fundamental points regarding the development and introduction of novel anti-TB agents. First, just because an agent is novel, the assumption that isolates are equally susceptible to this agent or that isolates with elevated MICs are rare does not apply in all settings [26, 48]. In fact, it has been recently demonstrated that Rv0678 mutations also confer elevated MICs to OPC-167832, which is currently in phase 1 and 2 trials, illustrating the value of elucidating the genetic basis of resistance [49]. Second, companion DST assays with breakpoints that are set based on modern microbiological principles are needed at the time approval of novel agents, as opposed to years later [39, 45, 50]. Third, if new drugs are used as part of weak regimens, resistance will inevitably evolve and may transmit rapidly. Consequently, molecular and phenotypic DST for BDQ, CFZ, and other drugs need to be scaled up, and further research on resistance mechanisms and the rates of pre-existing resistance in different settings is required, ideally, before novel regimens are introduced. In this context, targeted NGS assays or WGS could play a key role for reflex testing to rapidly rule-in resistance to the vast majority of drugs and enabling real-time surveillance of DR isolates [28, 29, 51].

## Supplementary Information

**Additional file 1: Table S1.** Origin of Rv0678 mutant isolates included in homoplastic analysis. **Table S2**. Drug resistance mutations and cluster analysis of collection 2 strains and EMBL-EBI ENA sequence read archive numbers. **Table S3**. BDQ and CFZ Minimum inhibitory concentration of 25 Rv0678 mutant and 12 wild type strains. **Table S4**. WGS of 273 isolates submitted to the EMBL-EBI ENA sequence read archive. **Table S5**. Epidemiological and genotyping data of 412 Mtbc-strains. **Table S6**. Phenotypic DST data of 412 Mtbc-strains, resistance mediating mutations for 273 whole genome sequenced isolates. **Table S7**. Drug resistance mediating mutations of all MDR clusters.

**Additional file 2.** Supplemental methods and description of resistance mutations.

**Additional file 3: Fig. S1.** Phylogenetic diversity and drug susceptibility of the 412 *M. tuberculosis* complex isolates from Eswatini. **Fig. S2**. Lineage distribution across Eswatini. **Fig. S3**. Isolates selected for further whole-genome sequencing analysis.

## Data Availability

Fastq files (raw sequencing data) for all strains analysed in this study are available from the EMBL-EBI European Nucleotide Archive sequence read archive (https://www.ebi.ac.uk/ena) [52] under the study IDs: PRJNA393767 [12], PRJEB20942 [13], PRJEB5280 [14], PRJNA395592 [15], PRJEB37777 [21], PRJEB6273 [22], PRJEB9680 [23], and PRJEB7281 [24], and details can be found in Additional file 1: Tables S1, S2 and S4.
