# Supplementary material for: MDR M. tuberculosis outbreak clone in Eswatini missed by Xpert has elevated bedaquiline resistance dated to the pre-treatment era
Source: Genome Med. 2020 Nov 25;12:104. doi: 10.1186/s13073-020-00793-8 (PMC7687760; doi:10.1186/s13073-020-00793-8)
Supplement: Supplementary file 2 — Additional file 2. Supplemental methods and description of resistance mutations. [file 13073_2020_793_MOESM2_ESM.docx]

**Supplemental methods and description of resistance mutations**

**Collection 2**

The thin layer agar (TLA) study was conducted at the Nhlangano Health Centre microbiology laboratory, Eswatini, by Médecins Sans Frontières-Switzerland and the Institute of Tropical Medicine (ITM), Antwerp, Belgium.

All consecutive patients tested in Nhlangano for presumptive TB, older than 15 years, who had not received TB treatment in the previous month and consented to be part the study, were included. Patients submitted two samples. Sample A was tested according to routine procedures with fluorescent microscopy, Cepheid GeneXpert MTB/RIF (G4), and cultured on both Löwenstein-Jensen (LJ) and TLA. If sample A was Xpert-positive, the sample B was sent to the National Reference Laboratory in Mbabane for culture and drug-susceptibility (DST) by MGIT and a third sample (C) was collected and tested on direct TLA DST for the study. If sample A was Xpert-negative, sample B was treated as sample A.

Direct DST on TLA was carried out for INH (0.2 and 1µg/ml), RMP (1 µg/ml), OFX (2 µg/ml), KAN (3 and 6µg/ml), MOX (2 µg/ml), LFX (1 µg/ml) and in addition, p-nitrobenzoic acid (500 µg/ml), RMP, to provide simultaneous identification of colony growth. One sector of the 4-quadrant plate contained PNB (the other sectors contained GC RMP and INH). Another plate was dedicated to the other drugs

Isolates from any positive TLA plate were sent to ITM to confirm the DST results using the proportion method on LJ for RMP (40 µg/ml) and INH (0.2, 1.0 µg/ml) and on 7H11 for KAN (6 µg/ml) OFX (2.0 and 8.0 µg/ml), LFX (1.0µg/ml) according to international guidelines.

Discordant results were resolved with the Hain Lifescience MTBDR*plus* V 2.0, Hain Lifescience MTBDR*sl* V 2.0, sequencing of target genes, or RMP minimum inhibitory concentration testing on LJ (10-160 µl/ml). In addition, whole-genome sequencing (WGS) was performed at the Borstel Supranational Reference Laboratory for all isolates that were resistant to RMP by at least one of the aforementioned approaches.

**Classical genotyping**

24-loci MIRU-VNTR typing and spoligotyping was done using standard approaches as described previously [1–3].

**Whole-genome sequencing analysis**

Libraries were prepared with the Nextera XT kit and sequenced using the Illumina MiSeq (251 and 301 bp, paired end), NextSeq 500 (151 bp, paired-end) and HiSeq 2500 (151 bp, paired end) according to the manufacturer’s instructions (Illumina, San Diego, CA, USA). The resulting reads were mapped to the *M. tuberculosis* H37Rv genome (GenBank ID: NC_000962.3) using the exact alignment tool SARUMAN and/or BWA [4]. For each strain, a coverage breadth of 99% relative to the reference genome was obtained, with an average coverage depth of at least 50-fold. WGS data was submitted to the EMBL-EBI ENA sequence read archive (Table S5).

From mapped reads, single nucleotide polymorphisms (SNPs) were detected using a minimum coverage depth threshold of 10 reads and a minimum allele frequency of 75% [5]. For the phylogenetic analysis, SNPs in resistance mediating genes and repetitive regions were excluded [6,7]. To avoid calling SNPs related to insertion and deletion artifacts, SNPs within a range of 12 nucleotides from each other were excluded. Positions that matched the threshold criteria for variant detection in 95% of all isolates were combined into a concatenated sequence alignment to calculate a pairwise distance matrix and phylogenetic tree. Isolates were grouped into potential transmission networks by agglomerative clustering with a maximum distance of 12 SNPs to the nearest neighbor [6,7].

A maximum likelihood tree (MLT) was calculated using the MetaPiga software v3.1[8] and the maximum likelihood ratio test [9]. Substitution models were tested with the general time reversible (GTR) substitution model superior to all other models [9]. Rate heterogeneity, without invariant sites using a gamma distribution as well as bootstrap resampling (1000 times) was applied and trees were calculated. Additional mid-point rooting using the FigTree software v1.4.2 and formatting using the online tool EvolView [10] was performed.

Individual concatenated SNP alignments of all lineages were used for a Bayesian coalescent analysis using the BEAST software v1.8.2 [11]. First, we compared different nucleotide substitution models under a coalescent constant size demographic prior that revealed a very strong support for GTR over Hasegawa, Kishino and Yano (HKY) substitution model (log10 Bayes factor >9). Other priors remained unchanged, i.e. discrete gamma distribution with four rate categories, a random starting tree, a tip date approach with a strict molecular clock (1x10^-7^ substitutions per nucleotide site per year) [5] and a uniform prior distribution. BEAST runs with chain lengths of 10,000,000 sampling every 1,000^th^ generation with a burn-in length of 10% were calculated. Analysis of BEAST log files, using the Tracer software v1.5, showed effective sample size (ESS) values above 200. Second, we tested different demographic models using the GTR substitution model with a tip dating approach with no significant differences (log10 Bayes factor <1), thus the simplest model, coalescent constant size, was selected for dating analysis. A maximum clade credibility tree (MCCT) was calculated using the TreeAnnotator software v1.8.2 to identify diversification events giving the time to the most common recent ancestor (TMRCA) with 95% highest posterior density interval (HPD).

**Comments on the area of technical uncertainty (ATU)**

Because of the inevitable variation in MIC testing of at least +/- one dilution, breakpoints cannot be set below the ECOFF, which is known as the CC in the TB field (i.e. otherwise gWT isolates would be misclassified as having elevated MICs) [12,13]. However, when WHO set the BDQ and CFZ CCs at 1 mg/L, it was noted that the MIC distributions of gWT strains and some Rv0678 mutants overlap (Fig. 3C), which means that some Rv0678 would inevitably show a poor reproducibility when tested at the CC (i.e. they would variably test susceptible and resistant because of the variation in testing alone) [14,15]. To minimise the misclassification of such strains as susceptible, EUCAST has recently introduced an ATU for some drugs, which corresponds to the concentration of the breakpoint in question (i.e. if MICs within an ATU are obtained, an isolate cannot be unambiguously classified as either susceptible or resistant). The ATUs for BDQ and CFZ, which are not currently recognised by the Clinical and Laboratory Standards Institute, EUCAST, or WHO, would correspond to 0.75-1 mg/L because of the non-standard concentrations tested in this study (i.e. the data that WHO reviewed did not include 0.75 mg/L, which is, therefore, equivalent to 1 mg/L [Fig. 3]) [12].

**Comments on validity of minimum inhibitory concentration data from this study**

No rigorously defined quality control ranges for H37Rv exist for either BDQ or CFZ, but the modes of the gWT distributions in this study (Fig. 3A and B) were comparable with the MIC data that WHO used to define the interim CCs for both agents [12,16]. Moreover, the upper ends of the gWT MIC distributions for both drugs (i.e. 0.75 mg/L) were equivalent to the interim CCs (i.e. WHO set 1 mg/L as the interim CCs given that this represented the tentative ECOFFs for both drugs when 0.75 mg/L was not included in the range of concentrations tested). Consequently, the reproducibility of testing in this study was deemed acceptable and there did not appear to be any detectable systematic shifts in the gWT MIC distributions in our data [12].

**Comment on non-wild type cut-off value (NCOFF)**

Valsesia et al. defined the resistant-population cut-off (RCOFF) as the “the largest inhibition zone diameter (or the lowest MIC) delineating a non-wild-type population” [17]. However, because a non-wild type population is not necessarily resistant, just as the wild type population is not necessarily susceptible, the term “resistant” is best avoided in this context. We have, therefore, opted to use NCOFF instead as a clinically neutral term.

**Resistance mediating and compensatory mutations**

The most common mutations found in *katG* and *rpoB* were the low fitness cost mutations resulting in S315T amino acid substitution (84%, 117/139 INH-resistant isolates) and S450L (47%, 60/127 RMP-resistant isolates; S531L *E. coli* numbering), respectively.

In GyrA, most mutations mediating FQ resistance (80%, 8/10 FQ-resistant isolates) were found at position 94 (D94G, D94N, D94Y) and 90 (A90V). The two amikacin and capreomycin-resistant isolates had the 1401 A/G mutation in *rrs*. Multiple variations in EthA and FabG1 were found (98%, 58/59 of all thionamide-resistant isolates).

References

1. Kamerbeek J, Schouls L, Kolk A, van Agterveld M, van Soolingen D, Kuijper S, et al. Simultaneous detection and strain differentiation of Mycobacterium tuberculosis for diagnosis and epidemiology. J Clin Microbiol [Internet]. 1997 [cited 2011 Apr 12];35:907–14. Available from: http://www.ncbi.nlm.nih.gov/pubmed/9157152

2. Supply P, Allix C, Lesjean S, Cardoso-Oelemann M, Rüsch-Gerdes S, Willery E, et al. Proposal for standardization of optimized mycobacterial interspersed repetitive unit-variable-number tandem repeat typing of Mycobacterium tuberculosis. J Clin Microbiol [Internet]. 2006 [cited 2011 Apr 12];44:4498–510. Available from: http://www.ncbi.nlm.nih.gov/pubmed/17005759

3. van Embden JD, Cave MD, Crawford JT, Dale JW, Eisenach KD, Gicquel B, et al. Strain identification of Mycobacterium tuberculosis by DNA fingerprinting: recommendations for a standardized methodology. J Clin Microbiol [Internet]. 1993 [cited 2011 Apr 12];31:406–9. Available from: http://www.ncbi.nlm.nih.gov/pubmed/8381814

4. Blom J, Jakobi T, Doppmeier D, Jaenicke S, Kalinowski J, Stoye J, et al. Exact and complete short-read alignment to microbial genomes using Graphics Processing Unit programming. Bioinformatics [Internet]. 2011 [cited 2012 Feb 2];27:1351–8. Available from: http://www.ncbi.nlm.nih.gov/pubmed/21450712

5. Roetzer A, Diel R, Kohl TA, Rückert C, Nübel U, Blom J, et al. Whole Genome Sequencing versus Traditional Genotyping for Investigation of a Mycobacterium tuberculosis Outbreak: A Longitudinal Molecular Epidemiological Study. Neyrolles O, editor. PLoS Medicine [Internet]. 2013 [cited 2013 Aug 7];10:e1001387. Available from: http://dx.plos.org/10.1371/journal.pmed.1001387

6. Comas I, Chakravartti J, Small PM, Galagan J, Niemann S, Kremer K, et al. Human T cell epitopes of Mycobacterium tuberculosis are evolutionarily hyperconserved. Nat Genet [Internet]. 2010 [cited 2012 Feb 2];42:498–503. Available from: http://www.ncbi.nlm.nih.gov/pubmed/20495566

7. Merker M, Barbier M, Cox H, Rasigade J-P, Feuerriegel S, Kohl TA, et al. Compensatory evolution drives multidrug-resistant tuberculosis in Central Asia. eLife [Internet]. 2018 [cited 2019 Mar 27];7. Available from: https://elifesciences.org/articles/38200

8. Helaers R, Milinkovitch MC. MetaPIGA v2.0: maximum likelihood large phylogeny estimation using the metapopulation genetic algorithm and other stochastic heuristics. BMC Bioinformatics [Internet]. 2010 [cited 2019 Aug 1];11. Available from: https://bmcbioinformatics.biomedcentral.com/articles/10.1186/1471-2105-11-379

9. Posada D, Crandall KA. MODELTEST: testing the model of DNA substitution. Bioinformatics [Internet]. 1998 [cited 2019 Aug 1];14:817–8. Available from: https://academic.oup.com/bioinformatics/article-lookup/doi/10.1093/bioinformatics/14.9.817

10. Zhang H, Gao S, Lercher MJ, Hu S, Chen W-H. EvolView, an online tool for visualizing, annotating and managing phylogenetic trees. Nucleic Acids Res. 2012;40:W569-572.

11. Drummond AJ, Rambaut A. BEAST: Bayesian evolutionary analysis by sampling trees. BMC Evol Biol [Internet]. 2007 [cited 2012 Jul 19];7:214. Available from: http://www.ncbi.nlm.nih.gov/pubmed/17996036

12. World Health Organisation. Technical Report on critical concentrations for drug susceptibility testing of medicines used in the treatment of drug-resistant tuberculosis [Internet]. Geneva; 2018. Available from: https://www.who.int/tb/publications/2018/WHO_technical_report_concentrations_TB_drug_susceptibility/en/

13. Mouton JW, Meletiadis J, Voss A, Turnidge J. Variation of MIC measurements: the contribution of strain and laboratory variability to measurement precision—authors’ response. Journal of Antimicrobial Chemotherapy [Internet]. 2019 [cited 2019 Aug 13];74:1761–2. Available from: https://academic.oup.com/jac/article/74/6/1761/5464306

14. Ismail NA, Said HM, Rodrigues C, Omar SV, Ajbani K, Sukhadiad N, et al. Multicentre study to establish interpretive criteria for clofazimine drug susceptibility testing. The International Journal of Tuberculosis and Lung Disease [Internet]. 2019 [cited 2019 Aug 13];23:594–9. Available from: https://www.ingentaconnect.com/content/10.5588/ijtld.18.0417

15. European Committee for Antimicrobial Susceptibility Testing. Area of Technical Uncertainty (ATU) in antimicrobial susceptibility testing [Internet]. 2019. Available from: http://www.eucast.org/fileadmin/src/media/PDFs/EUCAST_files/Breakpoint_tables/Area_of_Technical_Uncertainty_-_guidance_2019-1.pdf

16. Schön T, Matuschek E, Mohamed S, Utukuri M, Heysell S, Alffenaar J-W, et al. Standards for MIC testing that apply to the majority of bacterial pathogens should also be enforced for Mycobacterium tuberculosis complex. Clinical Microbiology and Infection [Internet]. 2019 [cited 2019 Aug 13];25:403–5. Available from: https://linkinghub.elsevier.com/retrieve/pii/S1198743X19300412

17. Valsesia G, Hombach M, Maurer FP, Courvalin P, Roos M, Böttger EC. The Resistant-Population Cutoff (RCOFF): a New Concept for Improved Characterization of Antimicrobial Susceptibility Patterns of Non-Wild-Type Bacterial Populations. Diekema DJ, editor. Journal of Clinical Microbiology [Internet]. 2015 [cited 2019 Aug 9];53:1806–11. Available from: http://jcm.asm.org/lookup/doi/10.1128/JCM.03505-14
