# Supplementary material for: MDR M. tuberculosis outbreak clone in Eswatini missed by Xpert has elevated bedaquiline resistance dated to the pre-treatment era
Source: Genome Med. 2020 Nov 25;12:104. doi: 10.1186/s13073-020-00793-8 (PMC7687760; doi:10.1186/s13073-020-00793-8)
Supplement: Supplementary file 3 — Additional file 3: Fig. S1. Phylogenetic diversity and drug susceptibility of the 412 M. tuberculosis complex isolates from Eswatini. Fig. S2. Lineage distribution across Eswatini. Fig. S3. Isolates selected for further whole-genome sequencing analysis. [file 13073_2020_793_MOESM3_ESM.pdf]

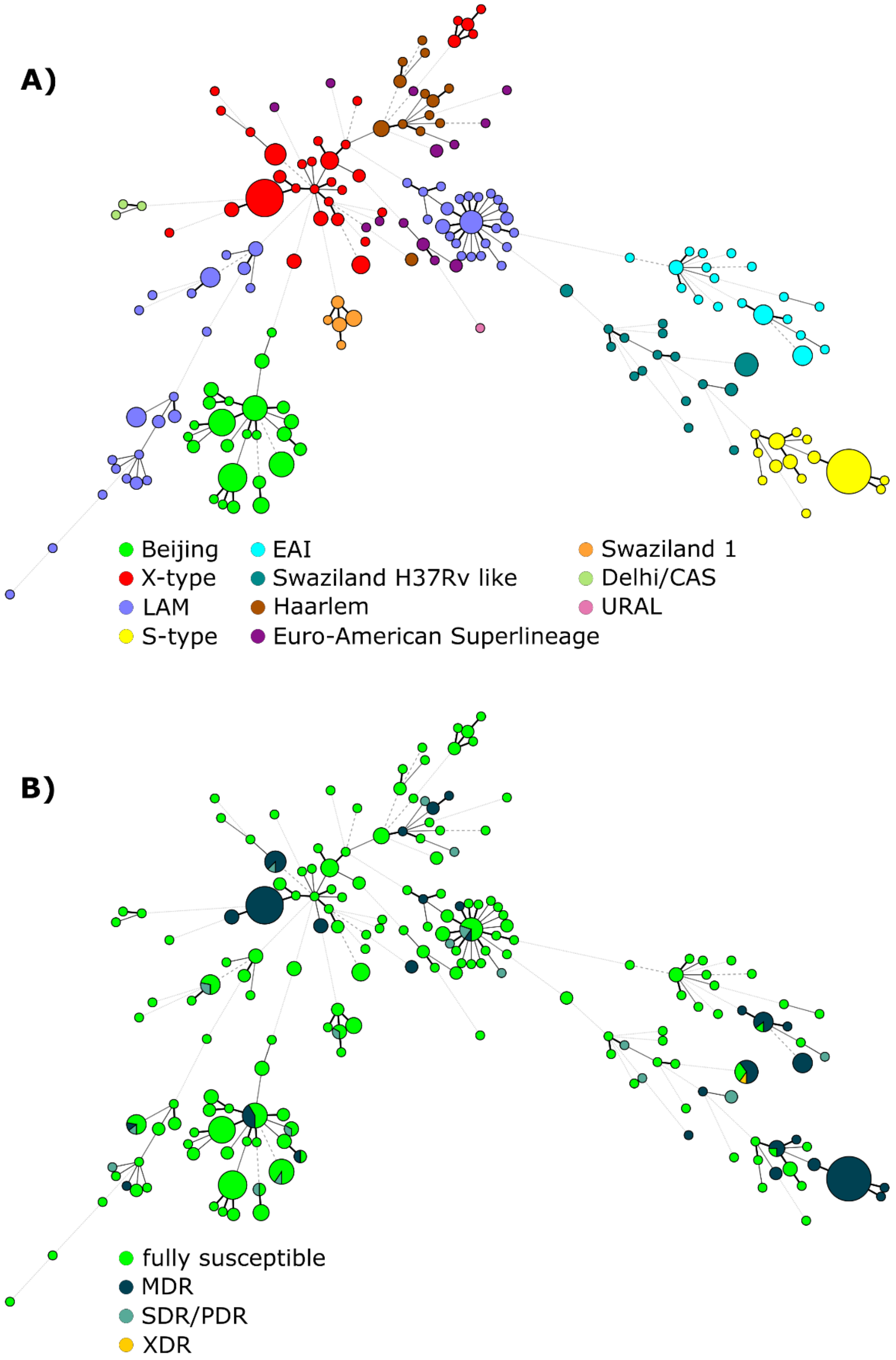


Fig S1. A) Phylogenetic diversity of the 412 *M. tuberculosis* complex isolates from Eswatini. Data are presented in a minimum spanning tree (MST) based on 24-loci MIRU-VNTR analysis. The relative size of the nodes represents the number of isolates belonging to a particular genotype (cluster, clone). Nodes are coloured according to the phylogenetic lineage classification defined in the figure. B) Drug susceptibility test results mapped onto the MST.

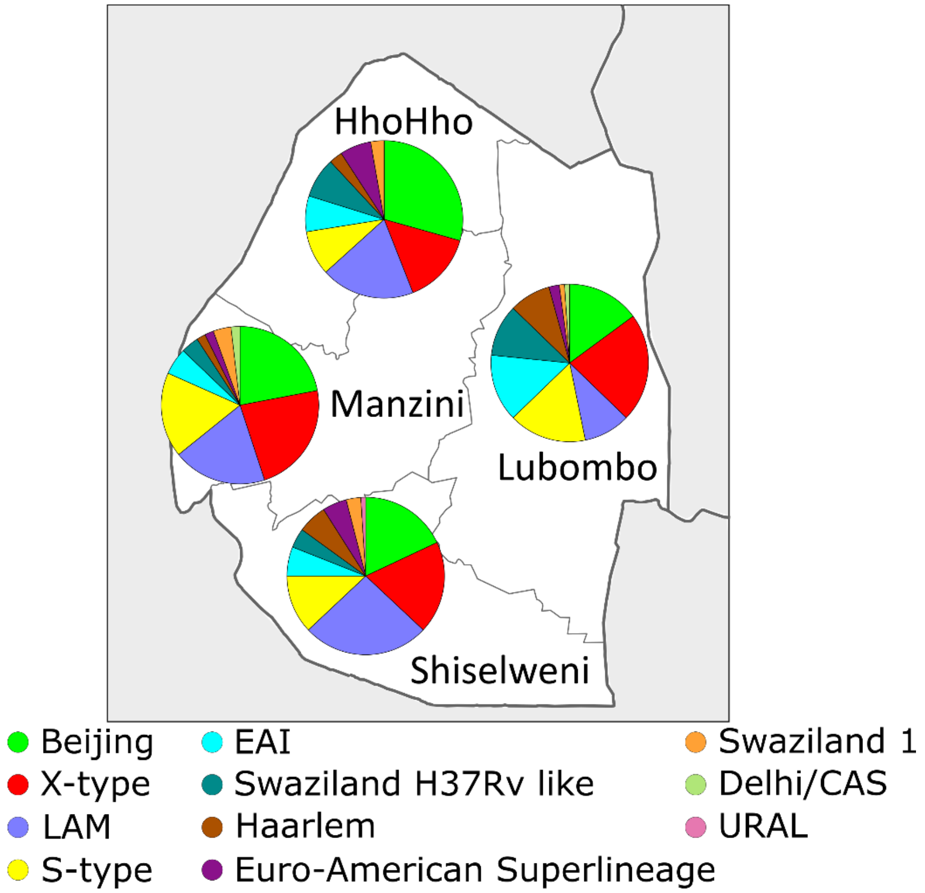


Fig S2. Lineage distribution across the four regions HhoHho, Manzini, Lubombo and Shiselweni included in the national survey 2009-2010.

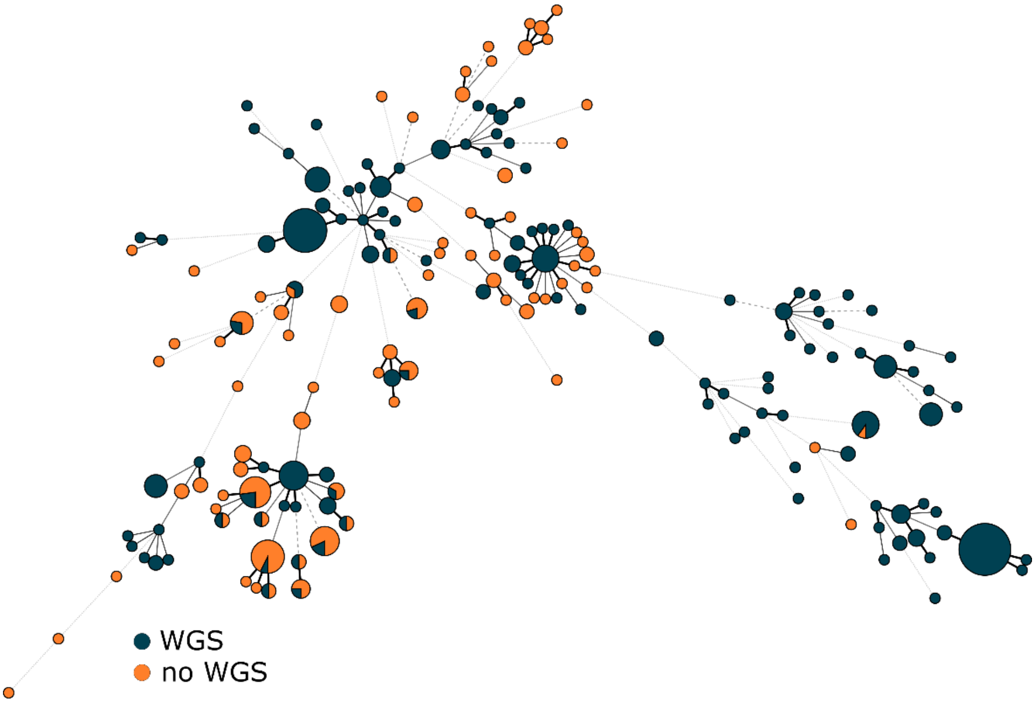


Fig S3. Minimum spanning tree (MST) based on 24-loci MIRU-VNTR highlighting the isolates selected for further WGS analysis.
